# Size and Content of the Sex-Determining Region of the Y Chromosome in Dioecious *Mercurialis annua*, a Plant with Homomorphic Sex Chromosomes

**DOI:** 10.3390/genes9060277

**Published:** 2018-05-29

**Authors:** Paris Veltsos, Guillaume Cossard, Emmanuel Beaudoing, Genséric Beydon, Dessislava Savova Bianchi, Camille Roux, Santiago C. González-Martínez, John R. Pannell

**Affiliations:** 1 Department of Ecology and Evolution, University of Lausanne, 1015 Lausanne, Switzerland; guillaume.cossard@unil.ch (G.C.); dessislava.savovabianchi@unil.ch (D.S.B.); camille.roux.1983@gmail.com (C.R.); santiago.gonzalez-martinez@pierroton.inra.fr (S.C.G.-M.); John.Pannell@unil.ch (J.R.P.); 2 Department of Biology, Jordan Hall, 1001 East Third Street, Indiana University, Bloomington, IN 47405, USA; 3 Faculty of Biology and Medicine, University of Lausanne, Bâtiment Génopode, 1014 Lausanne, Switzerland; emmanuel.beaudoing@unil.ch; 4 National Centre for Genomic Resources (CNRGV), 24 Chemin de Borde Rouge—Auzeville—CS52627, 31326 Castanet Tolosan Cedex, France; genseric.beydon@toulouse.inra.fr; 5 CNRS, University of Lille, UMR 8198—Evo-Eco-Paleo, F-59000 Lille, France; 6 BIOGECO, INRA, University of Bordeaux, 33610 Cestas, France

**Keywords:** bacterial artificial chromosomes (BAC), RNA sequencing (RNAseq), genetic map, transposable element, gene density

## Abstract

Dioecious plants vary in whether their sex chromosomes are heteromorphic or homomorphic, but even homomorphic sex chromosomes may show divergence between homologues in the non-recombining, sex-determining region (SDR). Very little is known about the SDR of these species, which might represent particularly early stages of sex-chromosome evolution. Here, we assess the size and content of the SDR of the diploid dioecious herb *Mercurialis annua*, a species with homomorphic sex chromosomes and mild Y-chromosome degeneration. We used RNA sequencing (RNAseq) to identify new Y-linked markers for *M. annua.* Twelve of 24 transcripts showing male-specific expression in a previous experiment could be amplified by polymerase chain reaction (PCR) only from males, and are thus likely to be Y-linked. Analysis of genome-capture data from multiple populations of *M. annua* pointed to an additional six male-limited (and thus Y-linked) sequences. We used these markers to identify and sequence 17 sex-linked bacterial artificial chromosomes (BACs), which form 11 groups of non-overlapping sequences, covering a total sequence length of about 1.5 Mb. Content analysis of this region suggests that it is enriched for repeats, has low gene density, and contains few candidate sex-determining genes. The BACs map to a subset of the sex-linked region of the genetic map, which we estimate to be at least 14.5 Mb. This is substantially larger than estimates for other dioecious plants with homomorphic sex chromosomes, both in absolute terms and relative to their genome sizes. Our data provide a rare, high-resolution view of the homomorphic Y chromosome of a dioecious plant.

## 1. Introduction

Most flowering plants are hermaphroditic or monoecious, but dioecy has evolved frequently and is found in about half of all plant families [1,2]. Although sex in many animals is determined by environmental triggers [3], in almost all dioecious plants studied so far it appears to be determined genetically (though see [4,5]), usually at a single genetic locus within a non-recombining sex-determining region (SDR) on a sex chromosome [6,7]. Many plant sex chromosomes are cytologically heteromorphic (currently known for 19 species in four families), but cytological differences between males and females are not evident in others (20 species in 13 families; reviewed in [7,8]). Indeed, closely related dioecious species may often differ in terms of their degree of heteromorphism [6,7]. We might expect the magnitude of the cytological difference between homologous sex chromosomes to increase with their age, as the result of the progressive genetic degeneration of the Y or W chromosome (in species with XY or ZW systems, respectively). However, although there is some evidence for this expectation [9], there are many exceptions. For instance, in *Coccinia grandis* (Cucurbitaceae), a species that evolved dioecy about three million years ago [10], the X and Y chromosomes are highly divergent, with a 10% elongation of the Y compared to the X. In contrast, the Y chromosome is smaller than the X in the palm genus *Phoenix*, in which the sex chromosomes may have diverged >50 million years ago [11,12,13].

Sex chromosomes are expected to diverge in length through the accumulation of repetitive sequences in the non-recombining chromosome (a phenomenon that appears to be particularly common in plants with heteromorphic sex chromosomes) or through the loss of genes [7]. Both processes can be attributed to the reduced efficacy of purifying selection in the SDR following suppressed recombination [14], which may have evolved because of advantages in linking the sex-determining locus with alleles that differentially benefit male or female fitness [15], or because of the accumulation of repetitive elements themselves [16]. Whereas the importance of sexually antagonistic selection in the evolution of suppressed recombination is plausible [15], there is still little empirical evidence for it. We also do not understand why X and Y (or Z and W) chromosomes of some species diverge rapidly, quickly becoming heteromorphic, while others remain homomorphic. We also remain largely ignorant about how the size of the SDR relates to its content, not only for species with putatively large SDR, such as *Silene latifolia* [17], but also for species with homomorphic sex chromosomes. Importantly, while species with homomorphic sex chromosomes may have an SDR that is small (even restricted to a single gene), their SDR might also be large. 

Characterization of the physical size of the SDR is challenging because of its often highly repetitive content. It is possible to compare the DNA content of males and females and attribute the difference to the sex chromosomes. As expected, such comparisons have revealed larger differences in species with heteromorphic sex chromosomes (see Supplementary Table S1, and [7]). However, to determine the actual size of the non-recombining SDR of the chromosome of the heterogametic sex (Y or W), it is necessary to identify markers on the sex chromosome, and to use them to build genetic maps that estimate the region that does not recombine in one sex, e.g., [18,19] (Supplementary Table S1). Alternatively, sex-linked markers may be used to identify, and then to sequence, bacterial artificial chromosomes (BACs) that contain long sections of the SDR [20], with subsequent assembly and potential chromosome-walking [21,22]. We take this approach in the current paper to assess the size and content of the SDR of the Y chromosome of the diploid dioecious plant *Mercurialis annua*. 

*Mercurialis annua* is a polyploid complex that shows striking variation in its sexual system, ranging from diploid populations that have fully separate sexes and a homomorphic XY sex-determination system, to androdioecy (where males co-occur with hermaphrodites) and monoecy [23,24,25,26]. The unusual variation in the sexual system of the *M. annua* complex lends itself to testing a number of hypotheses about the selection of combined versus separate sexes [27,28,29] and the evolution of sex determination and sex chromosomes [24,30]. Previous work, based on de novo sequencing, genetic mapping of SNPs from open reading frames (ORFs) segregating in crossing families, and genome capture from males and females sampled across the species range (representing about 7% of *M. annua* genome; [31]), has allowed the assembly of the diploid *M. annua* genome into eight linkage groups (corresponding to the 2n = 16 chromosomes of the diploid karyotype) and the identification of 568 sex-linked transcripts on the largest linkage group (i.e., chromosome 1), representing about 33% of the genes on the chromosome [30]. Genome and transcriptome analyses suggest that the SDR of *M. annua* is mildly degenerate, with a single gene interrupted by a premature stop codon and the accumulation of transposable elements and other repetitive DNA [30].

Here, we aimed (1) to estimate the physical length of the diploid *M. annua* SDR in relation to the size of the Y chromosome and to the rest of the genome, and (2) to characterize its content and genomic structure, including the identification of additional sex-linked genes that were either not previously mapped (due to the absence of suitable variation), or that were not among the ORFs used for previous mapping [30]. To estimate the size of the SDR, we identified male-specific polymerase chain reaction (PCR) products on ORFs, and used them to identify and sequence Y-linked BACs from two males. We inferred the minimum size of the SDR in terms of the size of the sex-linked region in the genetic map of *M. annua* [30] associated with the BACs. Our analysis suggests that the non-recombining SDR of the Y chromosome of *M. annua* is among the largest known for a homomorphic plant sex chromosome.

## 2. Materials and Methods

### 2.1. Overview

**Figure 1:**
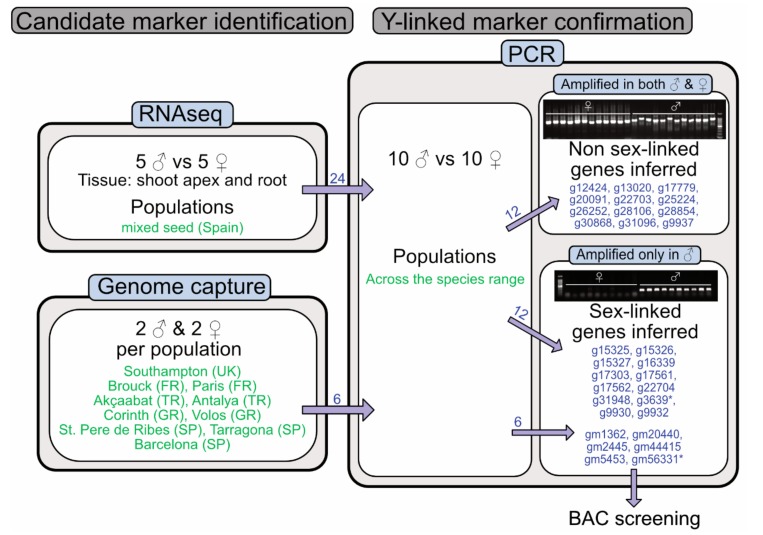
Graphical summary of diploid *Mercurialis annua* populations used, and the techniques we employed. Sampled populations are indicated in green, whereas polymerase chain reaction (PCR) products are indicated in blue. The PCR products marked with an asterisk represent the same sequence. The numbers next to the arrows indicate successful transcript attribution. The RNA sequencing (RNAseq) data come from [32], and the genome capture data from [31].

Our approach is summarized in Figure 1. We used male-specific gene expression [32] and male-specific genome sequences from a large sample of males and females of *M. annua*, sampled from across the species’ range [31], to identify potential Y-linked markers. We verified the Y-linked status of these markers by male-specific PCR amplification and used Sanger sequencing to verify that the amplified sequences were similar to the expected transcripts. We then used PCR reactions to probe a BAC library constructed from two diploid *M. annua* males, collected near Lausanne in Switzerland. Finally, we used long-read (Pacific Biosciences, Menlo Park, CA, USA) sequencing to assemble contigs of the identified Y-linked BAC sequences for subsequent analysis of content and genomic structure. 

### 2.2. Identification and Confirmation of Y-Linked Markers

We used two different approaches to identify Y-linked markers. First, we selected 24 genes that were expressed in at least two of five males and in none of five females in at least two sample types. Sample types were either roots or shoots, sampled either before or during flowering [32]. Of these 24 genes, 12 showed male-specific PCR amplification, based on an assay of ten males and ten females sampled across the species’ range (Figure 1). Additionally, we identified six more Y-linked markers on the basis of sequences that were obtained by genome capture from males, but not females, sampled from across the species range (details in [31]). These additional six sequences were also confirmed by PCR, as explained above. One of the six sequences identified by this second approach was identical to one of the 12 sequences identified on the basis of gene expression data. In total, therefore, we identified 12 + 6 − 1 = 17 sex-linked markers, which likely represent Y-specific transcripts, or parts thereof (Supplementary Table S2). All sequences were amplified with the same PCR program, which used a 55 °C annealing temperature, 1.5 min amplification time and 30 cycles. All sex-linked markers were Sanger-sequenced to confirm that PCR products were indeed the expected gene sequences (see Supplementary File S1). 

### 2.3. Bacterial Artifical Chromosome Library Construction, Sequencing and Assembly

*Mercurialis annua* leaves from two males were collected in November 2013 from wild plants growing on the campus of the University of Lausanne (Switzerland). Following storage of leaf material at −80°C, high molecular-weight DNA was obtained from these samples using nucleus extractions at the Centre National de Ressources Génomiques Végétales (CNRGV), Toulouse, France. The DNA was fragmented and ligated into BAC vectors (pIndigoBAC-5) before transformation. The resulting BAC library was screened for the presence of the 17 Y-specific PCR markers described above, and we selected 18 recombinant colonies that amplified targeted markers. DNA extracted from these colonies was sequenced at the Centre of Integrative Genomics, University of Lausanne, using Pacific Biosciences (PacBio) technology.

Specifically, the BAC DNA was sheared in a Covaris g-TUBE (Covaris, Woburn, MA, USA) to obtain fragments with a mean length of 6 Kb. After shearing, the DNA size distribution was checked on a fragment analyzer (Advanced Analytical Technologies, Ames, IA, USA). About 300 ng were obtained in 150 µL at 12,000 rpm or 13,684 ×g on a Heraeus Biofuge Fresco (Thermo Fisher Scientific, Waltham, Massachusetts, United States) and was then concentrated by SpeedVac (Thermo Fisher Scientific) to 4 µL. Barcoded adapters were added to each BAC during ligation, and BACs were pooled for sequencing. Multiplexing was performed using the SMRTbell Barcoded Adapter Prep Kit #100-465-800 (Pacific Biosciences). The sheared DNA (1.3 µg) was used to prepare each SMRTbell library with the PacBio SMRTbell Template Prep Kit 1 (Pacific Biosciences) according to the manufacturer’s recommendations. BAC DNA was also sheared to 30 Kb and 50 Kb, to obtain two follow-up libraries (pool1 and pool2), without multiplexing.

Each library was sequenced on one SMRT cell with P6/C4 chemistry and MagBeads on a PacBio RSII instrument (Pacific Biosciences) at movie lengths of 240 min for the multiplexed library and 360 min for the follow-up libraries. Assembly was performed using the PacBio module ‘RS_HGAP_Assembly.2’ in SMRTpipe version v2.3.0 (Pacific Biosciences). The multiplexed library assembly did not result in many circularized individual BACs, possibly because of the limited fragment length required for multiplexing. We therefore mixed equal quantities of DNA from the BACs, split into two independent pools (pool1, pool2), and we sequenced the corresponding long reads. We used the partial assembly of the multiplexed BAC library to identify reads that only mapped to BACs of known origin (from demultiplexing), and we assembled those longer reads independently. After successfully circularizing the longer read contigs, we removed the vector sequence and linearized the BAC contigs. We used Geneious v9 [33] to assemble these BAC contigs into scaffolds. We used the scaffolds, which contained an overlapping BAC sequence, for ORF counting, but the BAC contigs were used for all other analyses, because they allowed detection of subtle differences in their sequence. Table 1 shows a summary of the final assemblies used in downstream analysis, and the full annotated assembly is provided in Supplementary File S3.

### 2.4. Transcript and Transposable Element Annotation

RepeatMasker [34] was run on the genomic contigs and the BAC contigs, using the custom *M. annua* repeat library [30]. This allowed us to compare the repeat content of the BACs to that of the full genome, and to produce a masked BAC assembly. BAC 8, whose ORFs were not sex-linked, was treated separately. The *M. annua* transcriptome ORFs were aligned to the masked BAC assembly with BLAT, using option minIdentity 92 [35]. BLAT allows large gaps that are compatible with introns. The resulting gff file was used to identify the number of sex-linked ORFs that localize on the BACs. The gff file was also used as a hints file, along with the parameter ‘species = arabidopsis’ by the gene predictor Augustus v3.2.3 [36], which was also run on the masked BAC contigs, to identify sex-linked genes that were not expressed in the samples used for genetic map construction. We identified the best reciprocal blastn hit [37] (e-value cut-off of 10^−6^) per predicted transcript to obtain information on gene expression from the RNAseq experiment that was used to identify genes with male-specific expression [32], and another that used three pooled samples per sex, with ten individuals in each pool, to investigate gene expression in three developmental times before, and one after, flowering in apical meristems [32].

## 3. Results

### 3.1. Identification of Male-Specific Genes Based on Expression and Genome Capture Data

Twelve of the 24 genes that had male-specific expression were consistently amplified in males only (and are thus putatively Y-linked), whereas the remaining 12 genes amplified in both sexes (and are thus X-linked or autosomal). We also identified six genomic regions present on the Y-chromosome using genome capture data [31], by identifying sequences that were entirely missing from all females, but present in all males; one of these coincided with a sequence found on the basis of gene expression data, so that, in total, we obtained 17 male-specific PCR products. Three groups of these PCR products, containing seven transcripts, were already expected to be physically closely linked, based on their localization on the same genomic contig (g9930/g9932 and g15325/g15326/g15327, g17561/g17562). We thus conservatively estimate that there are 13 independent parts of the Y chromosome that can be sampled with these PCR products. Details for male-specific PCR amplification primers are summarized in Supplementary Table S2. Sequences of the products are provided in Supplementary File S1.

### 3.2. Bacterial Artifical Chromosome Assembly

Our screening approach allowed us to identify 17 BACs containing 11 independent Y-linked DNA sequences, which we confirmed via male-specific PCR. A further BAC (BAC 8) turned out to be a false positive, i.e., it did not contain Y-linked sequence. The BACs were aligned to each other and grouped into 11 non-overlapping sets. Four of these groups contained the same male-specific PCR product, indicating either sequence duplication or within-population variation (recall that the sequences were obtained from two different males from Switzerland). Finally, 10/11 independently localizing male-specific PCR products were found in these 11 non-overlapping BAC groups (Table 1). Overall, these 11 BAC groups cover a genomic region of 1.5 Mb, corresponding to about 0.47% of the haploid *M. annua* genome.

### 3.3. Functional Annotation of Genes Located on the Bacterial Artifical Chromosomes

**Figure 2:**
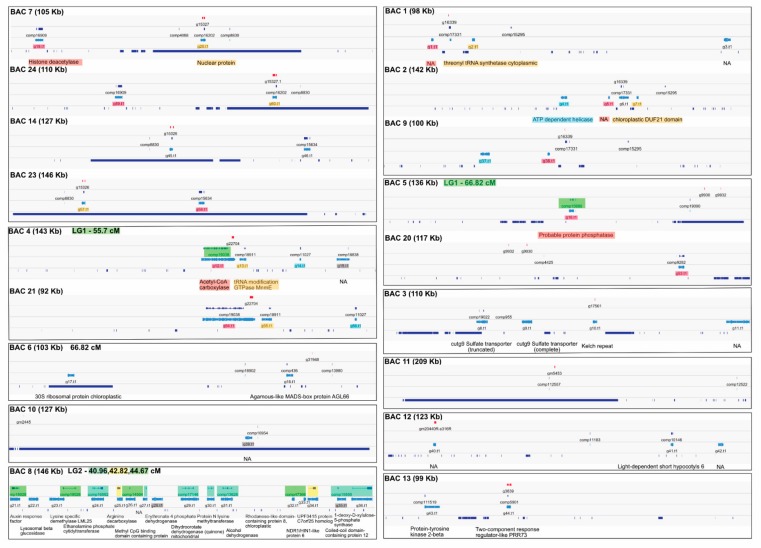
Graphical summary of the assembled BACs. Each assembly is annotated with the male-specific PCR product location (red), ORF *BLAT* hits (top dark blue), Augustus gene predictions on the repeat masked assembly (light blue), and BLAT hits from the *M. annua* repeat library (bottom dark blue). Each group of non-overlapping BACs is surrounded by a black box. Identical predicted gene models are indicated by overlaid colored boxes, for visual alignment of BACs containing the same genes. Green overlaid boxes indicate mapped ORFs that also map to the BACs, and their female recombination map position. Only genes with gray overlaid boxes were not expressed at all in the RNAseq experiments. The names displayed under the gene predictions are from their first blastp hit to the non-redundant (nr)protein database. Note the BAC contigs are not illustrated at the same scale.

By mapping the *M. annua* ORFs to the BAC scaffolds, we identified 24 broad genomic regions that each contained one or more complete ORFs (i.e., ORFs that mapped over their full length with identity >90%). Most of these ORFs can be considered putatively functional genes in the non-recombining region of the Y chromosome, both because they appear to be full-length genes, as well as because all but three of them had a best reciprocal blastn hit that was expressed in one or both of the expression experiments (the three unexpressed ORFs are marked in grey in Figure 2; expression data are available in Supplementary File S3). The exact gene number matching the broad 24 BAC regions is difficult to estimate because some ORFs overlap and may represent alternatively spliced variants of the same gene. Nevertheless, we identified at most 51 ORFs that mapped across their full length to the BACs, 14 of which blasted against sequences in the NCBI’s non-redundant (nr) nucleotide database. In addition, we found 87 ORFs that mapped over part of their sequence length to the BACs, and some of these may represent truncated genes on the Y-chromosome; these sequences were located in 53 broad BAC regions. Finally, we found one complete and one truncated copy of the same gene (a sulfate transporter) next to each other on BAC 3, suggesting that it might be the product of a localized, incomplete gene duplication. BAC localization of ORFs and gene annotation information is provided in Supplementary File S4.

Gene prediction using Augustus v3.2.3 identified a further 12 putative genes on the BAC sequence (Table 1) that did not overlap with previously identified ORFs. These are likely candidates for further male-specific PCR (full sequences are provided in Supplementary File S5). Some of the ORFs mapping to the sequenced BACs might be involved in either sex determination or male-beneficial effects and are good candidates for future study (see Table 1). They include a transcript in BAC 6 similar to the agamous-like MADS-box protein AGL66, which is required for pollen maturation and pollen-tube growth in *Arabidopsis* [38]. Another transcript, from BAC 12, is similar to light-dependent short hypocotyls 6, which is part of a family involved in response to light and organogenesis [39]. Finally, BAC 13 contained a transcript matching two-component response regulator-like PRR73, which controls photoperiodic flowering response [40]. Identification of such candidate genes is of course only the first step towards establishing involvement in sex determination and may be erroneous. For instance, BAC 8 revealed a strong candidate sex determiner (similarity to auxin response factor), but BAC 8 is probably not on the SDR, because its assembled sequence did not contain the male-specific PCR product used to identify the BAC, and most of its ORFs in fact mapped to an autosome (LG2; see Figure 2). Revealingly, BAC 8 also is very different from the other BACs in terms of both ORF density and repeat content (Figure 2). 

Four groups of BACs contained the same ORFs (Figure 2). For the group containing BACs 1, 2 and 9, we found that three similar, but different, genomic regions had been sequenced. BAC 2 contains a predicted gene not found in the other BACs (possibly a chloroplastic insertion, Figure 2). However, BAC 1 and BAC 9 also differ, because BAC 9 is missing a predicted gene present in BAC 1 and BAC 2. As only two males were sequenced, each with a single Y chromosome, the results can be interpreted as a duplication of the whole BAC sequence, or assembly error. The finding of putatively duplicated sequences of the Y is consistent with the expected accumulation of male-beneficial (and possibly sexually antagonistic) variants, or points to ongoing degeneration through repeat proliferation. For example, a duplicated sequence linked to the Y chromosome and associated with the origin of males has been found in date palm [41]. The remaining three groups of BACs in *M. annua* appear to have sampled the same genomic region multiple times.

### 3.4. Comparison of Transposable Element Density and Type Between the Bactertial Artificial Chromosomes and the Full Genome

Using RepeatMasker v 4.0.7 [34], we inferred that 76.9% of the BAC assembly comprised repetitive elements, substantially higher than the 47.9% repetitive content across the full genome. This was true for all categories of repetitive sequence, except for simple repeats. Long terminal repeats (LTRs) showed the highest enrichment on the BACs compared to the genomic contigs (25.33% vs. 8.45%; Supplementary Table S3). BAC 8 had a lower repeat content than that across the rest of the genome, increasing our confidence that it is indeed not sex-linked.

## 4. Discussion

### 4.1. Identification of Y-Linked Markers

Using a combination of RNAseq and genome-capture data, we have identified 17 new single or low-copy Y-linked markers in diploid dioecious *M. annua*, which we confirmed through male-specific PCR amplification. We note that 12 of 24 transcripts with male-specific expression could only be amplified in males and are thus Y-linked. However, the best reciprocal blastn hit of these transcripts was expressed in some female samples of the RNAseq experiment, typically for pooled individuals (Supplementary File S3). Possible explanations for expression in females of genes identified in our expression experiment as having male-specific expression include: (1) contamination of the female pools with male RNA; (2) similar transcripts from other parts of the genome producing the best reciprocal blastn hit; (3) sequence divergence between the X and Y copy of the transcripts, so that PCR primers only amplify the Y-linked copy; and/or (4) duplication and sequence divergence of some transcripts on the Y chromosome. We nevertheless infer that these PCR products represent Y-linked genes because those that could be mapped are located close to the extreme ends of the SDR, and none mapped to a different genomic region. Our results confirm that the X and Y chromosomes of *M. annua* are differentiated at the sequence level, even though they appear homomorphic (see below). Moreover, the successful search for new sex-linked transcripts on the basis of sex-limited expression suggests that ignoring or filtering sex-limited genes in transcriptome analysis may overlook loci in the SDR Muyle [42]. 

The identification of 17 new potential single or low-copy sex-linked markers in *M. annua* represents a substantial advance on previous work on the species by Khadka et al. [43], who identified a single-sequence characterized amplified region (SCAR) marker that was male-specific. This SCAR marker was later found to correspond to a high-copy transposable element that is present in both sexes [30,43], and is thus of limited utility beyond the sexing of pre-reproductive individuals (see [32]). We used several of the new sex-linked markers to probe a newly constructed BAC library for sex-linked genomic regions, which were the main focus of the present study. 

### 4.2. Size of the Sex-Determining Region

We may estimate the size of the SDR of *M. annua* using two different approaches that suggest rather different values. First, six ORFs contained in the BACs described here, which we independently inferred to be non-recombining based on the PCR result, were found in the sex-linked region of *M. annua* previously mapped by Ridout et al. [30]. These transcripts span a region from 52 to 66.82 cM in the female recombination map, corresponding to 441 ORFs, a length of 14.5 Mb and a proportion of 4.86% of the genome. This estimate is somewhat smaller than the one based on the mapping families alone (568 ORFs, equivalent to 19 Mb; [30]).

Second, given that only 6 of the 441 (1.3%) of sex-linked transcripts from Ridout et al. [30] mapped to the combined, non-overlapping, 1.5 Mb of the sequenced BACs, we might infer the SDR to be 1.5 Mb × 441/6, or about 110 Mb (assuming that the sex-linked transcripts are distributed similarly on the rest of the SDR not sampled by our BACs). This second estimate corresponds to about 34% of the haploid genome of *M. annua* and is evidently much too large. It would seem likely, therefore, that our BACs substantially under-represent the average gene density across the SDR. Alternatively, this view might suggest an over-estimate of the number of sex-linked genes from [30]. We thus suggest that the most reliable estimate is still based on the number of transcripts not recombining in males, i.e., between 14.5 Mb (the length spanned by the sex-linked BACs) and 19 Mb (inferred from recombination in small crossing families). 

Plant species with homomorphic sex chromosomes so far investigated have been found to have a SDR < 1% of the total length of the Y chromosome, e.g., *Vitis vinifera* [44,45], several *Populus* species [46,47], and *Fragaria chiloensis* [48]. *Mercurialis annua* thus appears to have a particularly large SDR for a species with homomorphic sex chromosomes. Ridout et al. [30] concluded that the sex chromosomes of *M. annua* have been evolving independently of the rest of the genome for some time, though still without substantial transcript degeneration. This view of only mild Y-chromosome degeneration is consistent with the fact that YY males, which lack an X chromosome, are completely viable in *M. annua* [49]. 

### 4.3. Content of the Sex-Determining Region

Although none of the de novo predicted genes on the BACs were obvious strong candidates for sex determination, three ORFs located on the BACs might function in sex determination or in the promotion of male function: a circadian gene involved in flowering (g44/g27235); a light response gene involved in organ and boundary differentiation (g41/g29576) [40]; and a transcription factor associated with pollen maturation (g18/g22596) [38]. These are interesting genes for future investigation, e.g., in surveys of population variation. Expression of these genes in two RNAseq datasets is presented in Supplementary Figure S1. None is significantly sex-biased. The pollen-maturation gene has higher expression in females early in development, but in males later in development. Similarly, the gene g27235, involved in flowering, was consistently more expressed in males than females in apical meristems, but showed an increased expression in mature leaves of males compared to females during flowering. A similar pattern was observed for the auxin response gene g21/g20779, which mapped to BAC 8 (i.e., it was not sex-linked), pointing to possible trans-regulation of the auxin response gene by the (unknown) Y-linked sex determiner. 

Analysis of the content of the BAC contigs in *M. annua* has revealed typical features of non-recombining sex chromosomes, congruent with expectations of partial Y-chromosome degeneration. For instance, the sex-linked BACs contained mainly repetitive elements, which are expected to accumulate in non-recombining regions [50]. Comparison with the genomic scaffolds [30] revealed that the BACs are enriched in transposable elements (TEs), a finding similar to that reported for the *Carica papaya* Y^h^ chromosome, where more than 80% of the non-recombining region on the Y chromosome comprises repetitive elements compared with only around 60% for the X chromosome [51]. We also found a clear case of partial gene duplication physically close to its complete gene copy, as well as multiple complete ORFs that did not completely map to the BACs. Although we have not been able to determine how many of these ORFs are bona fide disrupted genes (because no X-only contigs are yet available), their high number is consistent with early stages of Y degeneration in *M. annua*. 

Finally, the overall gene density in the sex-linked BACs of *M. annua* appears to be substantially lower than that observed in the rest of the genome [30], consistent with the much higher gene density and a much lower repeat composition found for BAC8, which turned out not to be sex-linked, compared with the sex-linked BACs or the sex-linked genomic contigs [30]. 

## 5. Conclusions

Dioecious plants offer tremendous scope for examining the evolution of sex chromosomes because separate sexes have evolved independently, often relatively recently. A particularly noteworthy feature of dioecious plants is the degree to which they vary in the relative sizes of their X and Y (or Z and W) chromosomes, with species that have strongly heteromorphic sex chromosomes often closely related to species whose sex chromosomes are homomorphic [6,7]. Species with homomorphic sex chromosomes might simply be young, or might be subject to processes that maintain relative uniformity between homologues and a small SDR, such as frequent turnover [52] or occasional recombination [53]. Our study of the Y chromosome of *M. annua* illustrates that the SDR in homomorphic sex chromosomes may also be relatively large. Indeed, our estimate of the SDR of *M. annua* is the largest for any plant species with homomorphic sex chromosomes studied so far, whether viewed in absolute terms, or relative to the size of the sex chromosomes or the rest of the genome. The BAC sequences analyzed here point to an SDR with low gene density and enriched for repeats, with often incomplete mapping of complete ORFs (see also [30]). In this sense, *M. annua* may represent a species at a particularly interesting intermediate stage along the path towards Y-chromosome degeneration and sex-chromosome heteromorphism. 

## Figures and Tables

**Table 1:** Bacterial artificial chromosome (BAC) contig information. Overlapping BACs are displayed in the same row, and their approximate cumulative single copy length is shown. Inference of potential candidate sex-determining genes is based on the description of the first blastp hit. The predicted gene, transcript ID in expression data and NCBI sequence ID precede the gene description.

BAC Contig	Length (Kb)	Male-Specific PCR Hit	ORFs	Additional Predicted Genes	Potential Candidate Genes for Sex Determination
**1, 2, 9**	170	g16339	3	3	
**3**	110	g17561	1	3	
**4, 21**	143	g22704	4	0	
**5, 20**	200	g9930, g9932	3	0	
**6**	103	g31948	3	1	g18/g22596: XP_021600590.1 Agamous-like MADS-box protein AGL66
**8**	146	NA	13	3	g21/g20779: XP_002519813.1 Auxin response factor
**10**	127	g2445	1	0	
**11**	209	g5453	2	0	
**12**	123	gm20440	2	2	g41/g29576: XP_002267312.1 Light-dependent short hypocotyls 6
**13**	99	g3639	2	0	g44/g27235: XP_021670876.1 Two-component response regulator-like PRR73
**7, 14, 23, 24**	215	g15326, g15327	5	0	

PCR: Polymerase chain reaction; ORF: open reading frame; NA: not available.

## Supplementary Materials

The following materials are available online at http://www.mdpi.com/2073-4425/9/6/277/s1. Figure S1: Plots of transcript per million data of genes localizing to the BACs and potentially involved in sex determination, in the two RNAseq experiments. Table S1: Summary of information on size and divergence in systems with homomorphic and heteromorphic sex chromosomes. References [10,17,18,19,22,45,46,47,48,50,51,54,55,56,57,58,59,60,61,62,63,64,65,66,67,68,69,70,71,72,73,74,75,76,77,78,79,80,81,82,83,84,85,86,87,88,89,90,91] are cited in Supplementary Table S1. Table S2: Information on PCR primers. Table S3: Summary output from RepeatMasker using the *M. annua* repeat library. File S1: Sanger sequence of PCR products. File S2: BAC assembly and gene prediction gff. File S3: Expression data of the BAC ORFs. File S4: BLAST information on ORF hits to the BAC assembly. File S5: Aminoacid sequence of predicted genes on the BACs.
