# Supplementary material for: Size and Content of the Sex-Determining Region of the Y Chromosome in Dioecious Mercurialis annua, a Plant with Homomorphic Sex Chromosomes
Source: Genes (Basel). 2018 May 29;9(6):277. doi: 10.3390/genes9060277 (PMC6027223; doi:10.3390/genes9060277)
Supplement: Supplementary file 1 [file genes-09-00277-s001.zip › genes-296661-supplementary.docx]

Supplementary Materials: Size and Content of the Sex-Determining Region of the Y Chromosome in Dioecious *Mercurialis annua*, a Plant with Homomorphic Sex Chromosomes

Paris Veltsos, Guillaume Cossard, Emmanuel Beaudoing, Genséric Beydon, Dessislava Savova Bianchi, Camille Roux, Santiago C. González-Martínez and John R. Pannell

1. Supplementary Figures


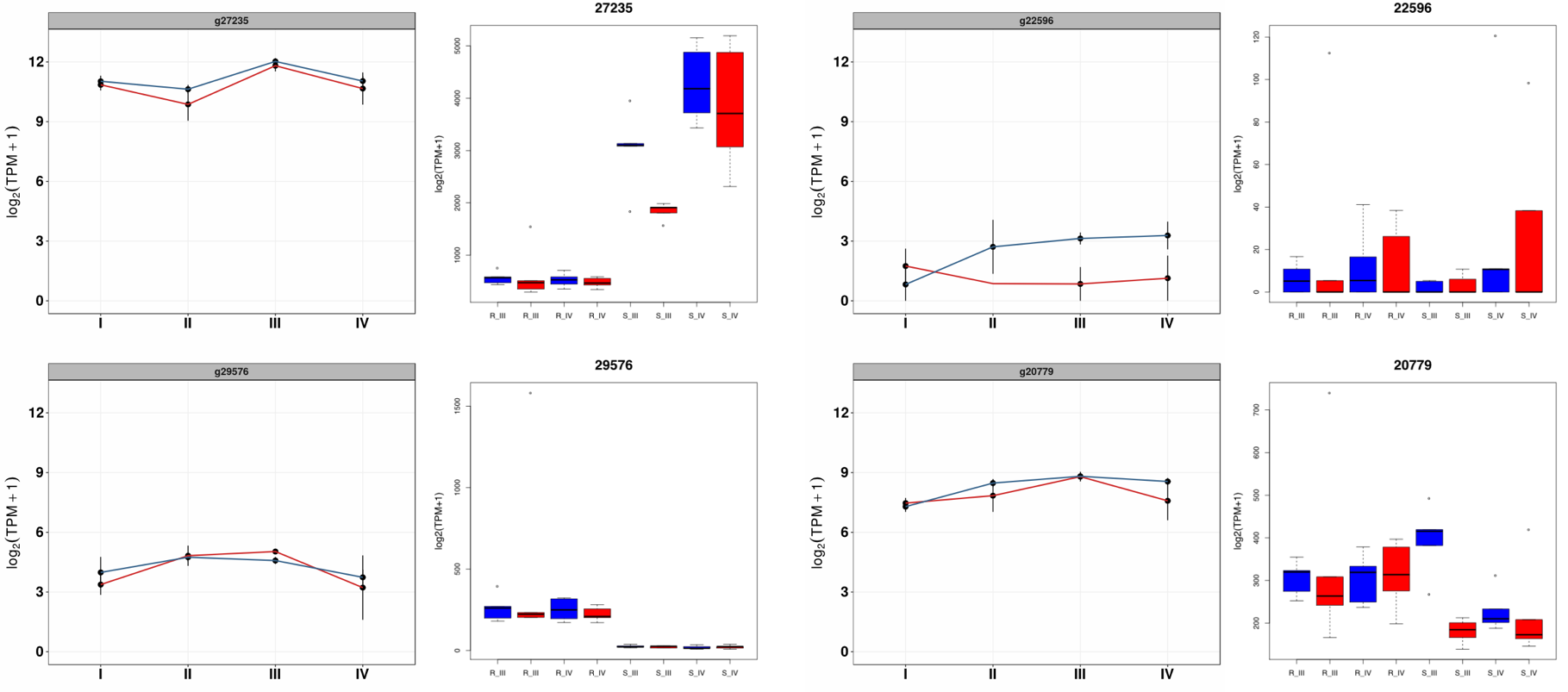


**Supplementary Figure S1.** Plots of transcript per million data of genes localizing to the BACs and potentially involved in sex determination in the two RNAseq experiments. The line plots show 3 stages before (I, II, III) and after (IV) flowering, and the boxplots show expression in roots (R) and shoots (S) before (III) and after (IV) flowering. Male and female expression are indicated in blue and red, respectively. None of the stages showed a statistically significance in sex bias in the RNAseq analyses generating these data. Data are taken from [31].

2. Supplementary Tables

**Supplementary Table S1.** Summary of information on size and divergence in systems with homomorphic and heteromorphic sex chromosomes.

| **Species** | **Estimated Haploid Genome Size in Mbp (Mean Male/Female)** | **Sex Chromosome Types** | **Heteromorphic Sex Chromosomes?** | **Estimated Size of Sex Chromosomes (Mbp)** | **Size Information on the Heteromorphic Sex-Specific Region (Mbp)** | **Notes** | **References** |
| --- | --- | --- | --- | --- | --- | --- | --- |
| **Angiosperms** | | | | | | | |
| *Cannabis sativa* | 817 (2n = 20) ^a^ | XX/XY | Yes (Y>X) | - | Diff M/F = c.a 47 | Difference of genome size between males and females | [54,55] |
| *Hulmulus lupulus* | 2836 (2n = 20) ^a^ | XX/XY | Yes (Y<X) | Y: 186.8 | Diff M/F = c.a. 73.4 | Difference of genome size between males and females | [56,57] |
| *Humulus japonicus* | 1569 (2n = 16/17) ^a^ | XX/XY_1_Y_2_ | Yes (Y>X) | Y_1_: 259.2  Y_2_: 238.6  X: 270.9 | Diff M/F = c.a. 307.1 | Difference of genome size between males and females | [56] |
| *Silene latifolia* | 2832 (2n = 24) ^a^ | XX:XY | Yes (Y>X) | 350 (X)  524(Y) | 349 | Non-recombining region estimated to be 2/3 of chr. Y | [17,58,59] |
| *Coccinia grandis* | 438 (2n=24) ^a^ | XX/XY | Yes (Y>X) | - | Diff M/F = c.a. 98 | Difference of genome size between males and females | [10] |
| *Rumex acetosa* | 1614 (2n = 14/15) ^a^ | XX/XY_1_Y_2_  (X/A sex determination) | Yes (Y_1_/Y_2_<X) | - | Diff M/F = c.a. 489 | Difference of genome size between males and females | [59,60] |
| *Rumex hastatulus*  *(1) Texas*  *(2) North Carolina* | (1) 1864 (2n=10) ^a^  (2) 1801 (2n = 8/9) ^a^ | (1) XX/XY  (2) XX/XY_1_Y_2_ | (1) Yes (Y>X)  (2) Yes (Y_1_/Y_2_<X) | - | (1) Diff M/F = c.a 349.1  (2) Diff M/F = c.a 333.5 | Difference of genome size between males and females | [61–63] |
| *Actinidia chinensis* | 758 (2n = 58) ^b^ | XX/XY | No | - | 5 | Fine scale mapping | [18,64,65] |
| *Asparagus officinalis* | 1323 (2n = 20) ^a^ | XX/XY | No | - | 1–10 | Raw estimate | [22,48,60] |
| *Phoenix dactylifera* | 670 (2n = 36) ^b^ | XX/XY | No | - | 5–13 | Mapping of sex-specific scaffolds | [19,66,67] |
| *Pistacia vera* | 585 (2n=30) | ZZ/ZW | No | 50 (largest chromosome pair) | 28 RAD reads with W allele | Sex linked RAD SNPs | [68] |
| *Carica papaya* | 372 (2n = 18) ^b^ | XX/XYY^h^ | No | Y^h^: 81 | 8.1 on Y^h^ (10 % of chr. Y^h^) | BAC sequencing of Y^h^ | [51,69] |
| *Fragaria virginiana (subdioecious)* | 782 (2n = 8x = 56) ^a^ | ZZ/ZW | No | - | No non-recombining region | Gentic mapping | [70] |
| *Fragaria chiloensis* | 400 (2n = 56) | ZZ/ZW | No | - | 0.280 | QTL and amplicon mapping | [48] |
| *Dyospyros lotus* | 1809 (2n = 30) ^b^ | XX/XY | No | - | ~1 | Assembly of Y-specific contigs | [71,72] |
| *Populus deltoides x nigra*  *Populus alba* | 480 (2n = 38) ^a^ | ZZ/ZW | No | - | 0.71 | Fine scale mapping | [46,73] |
| *Populus trichocarpa x tremuloides*  *P. nigra*  *P. balsamifera* | 485 (2n = 38) ^a^ | XX/XY | No | - | 0.1 (*P. trichocarpa; P. balsamifera)* | Identifiation of sex-associated SNPs | [47,74] |
| *Salix virminalis*  *S. purpurea* | 450 (2n = 38) ^a^ | ZZ/ZW | No | - | <2.5 (*S. purpurea*) | Fine scale mapping of SD locus | [75] |
| *Vitis vinifera* | 487 (2n = 38) ^b^ | XX/XY | No | - | 0.143–0.155 | Estimated to be less than 1 % of chr. Y | [44,45,76] |
| *Mercurialis annua* | 645 (2n = 16) | XX/XY | No | - | 14.5–19 | BAC sequencing | [77]; This study |
| **Vertebrates** | | | | | | | |
| *Homo sapiens* | 2900 (2n = 46) ^b^ | XX/XY | Yes (Y<X) | X = 160  Y = 60 | 57 | Estimated to be 95% of chr. Y | [78,79] |
| *Pan troglodytes* (Chimpanzee) | 2700 (2n = 48) ^b^ | XX/XY | Yes (Y<X) | - | 25.8 | BAC sequencing of chr. Y | [78,79] |
| *Gallus gallus* (Chicken) | 1050 (2n = 78) ^b^ | ZZ/ZW | Yes (W<Z) | Z: 82  W: 55 | Very low | Observations of chiasmata during meiosis | [80,81] |
| *Struthio camelus* (Ostrich) | 1230 (2n = 80) ^b^ | ZZ/ZW | No | 95.6 | 31.9 (~1/3 of Z chr.) | Estimated to be a third of the Z chromosome | [82,83] |
| *Gasterosteus aculeatus* (Threespine stickleback) | 530 (2n = 42) ^b^ | XX/XY | No (different centromere positions) | X: 20.2  Y: 13.2 | 10 | BAC sequencing and FISH | [84] |
| *Oryzias latipes* (Medaka) | 700 (2n = 48) ^b^ | XX/XY | No | 33.7 | 3.4 | Estimated to be 10% of chr. 1 | [85] |
| **Insects** | | | | | | | |
| *Drosophila melanogaster* | 360 (2n = 8) ^b^ | XY  (X/A sex determination) | Yes (Y<X) | X: 41.8  Y: 40.9 | 40.9 | 100 % of neo-Y | [50,86] |
| *Drosophila albomicans* | 366 (2n = 6) ^b^ | Neo-XY | Evidence for degeneration | 73.2 (about 40% of the genome | 73.2 | 100 % of neo-Y | [50,87] |
| *Drosophila pseudoobscura* | 312 (2n = 10) ^b^ | Neo-XY | Evidence for degeneration | - | - | 100 % of neo-Y | [50,88] |
| *Drosophila miranda* | NA (2n = 9/10) | Neo-XY | Yes (Neo-Y<Neo-X) | ~4.3 Mb (of coding sequence) | ~4.3 Mb of coding sequence | 100 % of neo-Y | [89,90] |
| **Flatworms** | | | | | | | |
| *Schistosoma mansoni* | 300 (2n = 16) ^c^ | ZZ/ZW | No | Z: 60.7  W: 60–70 | 26.7 (44%) | Estimated to be 44% of chr. Y | [91] |

^a^ genome size estimated by flow cytometry; ^b^ genome size estimated from sequencing data – haploid genome size; ^c^ genome size estimate used in study, without reference citation.

**Supplementary Table S2.** Information on primers, amplifying ORFs in males only or both sexes. Names indicated by a * amplify the same transcript.

| **Name** | **Primer_F** | **Primer_R** | **PCR Amplification** | **Identification Method** |
| --- | --- | --- | --- | --- |
| **g15325** | CATTGGCAGTGAAACCCTGG | TGGATTTCAGTGCAAAGCCT | Male | RNAseq |
| **g15326** | GTGACTCTCTCCCTATGGCC | AAACCTTTCTGCACGAGTCG | Male | RNAseq |
| **g15327** | TTTGTTGCACCCCGATCAAG | CATCCTCCCTTGCAACGTTT | Male | RNAseq |
| **g16339** | ATTCGGGTTTCTCGAGTGGT | ACTAACTGTGTACCAAAAGCTTG | Male | RNAseq |
| **g17303** | GTGCGGCAGTCAACACTAC | GACCGGGCTTGAAGTTGAAG | Male | RNAseq |
| **g17561** | TCCAGTCATCCCAACGTTCA | TGAACAGAAGGCAGAGACGA | Male | RNAseq |
| **g17562** | ACAGTCGGCCTTCATCTTCA | TGAGTCAGAAGAAGAACAAGCT | Male | RNAseq |
| **g22704** | TCCGGGAAGCCAGAAATAGT | CGAAGCCCATCCATCAACTG | Male | RNAseq |
| **g31948** | TGGAGACGATGGATGTTGCT | AACAGACGGCTCACCCATC | Male | RNAseq |
| **g3639*** | ACTGCTGGGACTATCACCTC | TGCATTCGAAGGAGTTTGGAC | Male | RNAseq |
| **g9930** | TGCTGGAAATGATGGTTGCC | ACAACTTCTCTCCAGCTGCT | Male | RNAseq |
| **g9932** | TGCTGGAAATGATGGTTGCC | ACAACTTCTCTCCAGCTGCT | Male | RNAseq |
| **gm1362** | AGGACGTTGTAGAGGTAGACC | GATGGGTCGACATAAGGCAT | Male | Exon capture |
| **gm20440** | GGTGTAGCCTTCCCCTTCTT | ACCACTGCCCTGAGAGAATC | Male | Exon capture |
| **gm2445** | CTAGTTGGAAGTTGGCGTGG | CCCTTTGCCAAACCGTGTAA | Male | Exon capture |
| **gm44415** | AAGTGTCGGCAGTCTTAGGT | GCCTCCATCATGAAGGCTTT | Male | Exon capture |
| **gm5453** | AAGCTTGTCCAGGGTCGTAG | CCTTTCAGAAGCAGCATTATGGA | Male | Exon capture |
| **gm56331*** | TCACTACTAGCACAGCCACC | CTGAGAGTTGAGGTTGCACAG | Male | Exon capture |
| **g12424** | GCGTGTGAGTGGGCTAATAG | GCACACCATTTTCTTCCTCCT | Both | RNAseq |
| **g13020** | TTGATCGGAGCAGAGAGTGG | GGTGTAGCCTTCCCCTTCTT | Both | RNAseq |
| **g17779** | TCCTGTTCTGACTTCGACGT | CGAAGAGGCCATGTTAAATCCA | Both | RNAseq |
| **g20091** | ATTGAGGAGCTTGTGGACCC | AGTGTCATGACTGGGTTCCC | Both | RNAseq |
| **g22703** | TCGCCTACTAGCCATGTTGT | AGAAAAGAAGAAGCCAGCCTG | Both | RNAseq |
| **g25224** | GCGACCAAAAGAGGCAGAAT | TGTTGCTGCTATCATCGTGC | Both | RNAseq |
| **g26252** | TCCCGATTCCTTCCAGTGAA | TGTTACGTATAGGGCAGCCA | Both | RNAseq |
| **g28106** | GGCTGGAATTGCTTTGAACG | TCAATTTGTGGACGCAGCAA | Both | RNAseq |
| **g28854** | TGGGGCATACTGATTTGATGTG | CTTCTGAGCTTCTGTCACCTT | Both | RNAseq |
| **g30868** | AAGAGTTTGAGGCTGCATCC | GGCTAATACACATGCGGTAGG | Both | RNAseq |
| **g31096** | GGTAATCCAGCTTCAGTGTGC | ACCACAGGAATCGATTGCAG | Both | RNAseq |
| **g9937** | TGGAGGATTATCATGTTGCAAAG | AGCCTCCTGATTCGACAACA | Both | RNAseq |

**Supplementary Table S3.** Summary output from RepeatMasker using the *M. annua* repeat library.

|  | | **Percentage of Sequence** | | | **Number Elements** | | | **Length (bp)** | | |
| --- | --- | --- | --- | --- | --- | --- | --- | --- | --- | --- |
|  |  | **Y BACs** | **BAC 8** | **Genome** | **Y BACs** | **BAC 8** | **Genome** | **Y BACs** | **BAC 8** | **Genome** |
| SINEs | Other | 0 | 0 | 0.03 | 0 | 0 | 1127 | 0 | 0 | 163,941 |
|  | ALUs | 0 | 0 | 0 | 0 | 0 | 0 | 0 | 0 | 0 |
|  | MIRs | 0 | 0 | 0 | 0 | 0 | 0 | 0 | 0 | 0 |
| LINEs | Other | 4.8 | 4.28 | 2.66 | 134 | 7 | 33,781 | 101,114 | 6295 | 14,537,810 |
|  | LINE1 | 2.87 | 2.22 | 2.01 | 64 | 5 | 22,157 | 60,373 | 3264 | 10,979,834 |
|  | LINE2 | 0 | 0 | 0 | 0 | 0 | 0 | 0 | 0 | 0 |
|  | L3/CR1 | 0 | 0 | 0 | 0 | 0 | 0 | 0 | 0 | 0 |
| LTR elements | Other | 26.63 | 4.61 | 8.45 | 708 | 14 | 129,707 | 560,624 | 6782 | 46,164,934 |
|  | ERVL | 0 | 0 | 0 | 0 | 0 | 0 | 0 | 0 | 0 |
|  | ERVL-MaLRs | 0 | 0 | 0 | 0 | 0 | 0 | 0 | 0 | 0 |
|  | ERV_classI | 0 | 0 | 0 | 0 | 0 | 51 | 0 | 0 | 13,842 |
|  | ERV_classII | 0 | 0 | 0.01 | 0 | 0 | 94 | 0 | 0 | 32,526 |
| DNA elements | Other | 6.49 | 4.01 | 2.65 | 254 | 24 | 63,665 | 136,533 | 5900 | 14,467,832 |
|  | hAT-Charlie | 0 | 0 | 0 | 0 | 0 | 0 | 0 | 0 | 0 |
|  | TcMar-Tigger | 0 | 0 | 0 | 0 | 0 | 0 | 0 | 0 | 0 |
| Unclassified | | 38.14 | 26.72 | 31.14 | 2605 | 182 | 813,812 | 803,031 | 39,296 | 170,128,904 |
| Total interspersed repeats | | 76.06 | 39.62 | 44.93 |  |  |  | 1,601,302 | 58,273 | 245,463,421 |
| Small RNA | | 0.1 | 0 | 0.06 | 6 | 0 | 1,397 | 2064 | 0 | 352,034 |
| Satellites | | 0 | 0 | 0 | 0 | 0 | 0 | 0 | 0 | 0 |
| Simple repeats | | 0.67 | 0.83 | 2.88 | 275 | 28 | 99,695 | 14,158 | 1228 | 15,743,639 |
| Low complexity | | 0.25 | 0.34 | 0.15 | 94 | 9 | 16,600 | 5336 | 505 | 838,784 |
| Bases masked | | 76.91 | 40 | 47.85 | 1,619,127 | 60,006 | 261,415,926 | 1,619,127 | 60,006 | 261,415,926 |

3. Supplementary files:

**Supplementary File S1.** Sanger sequence chromatograms of single copy PCR products.

**Supplementary File S2.** BAC assembly sequences and mapping files (gff) used to produce Figure 2., to be used in a genome browser for interactive exploration of the assemblies.

**Supplementary File S3.** Expression data of the BAC and PCR genes in the two RNAseq experiments.

**Supplementary File S4.** Blast information on ORF hits to the BAC contigs.

**Header**

**Name**—ORF name

**Annotation**—Blastn information on ORFs whose length fully maps to a BAC

**type**—Information on whether the ORF represents a complete transcript

**length_nt**—The length of the ORF in basepairs

**sum_hits**—The length of all BLAST hits of a given ORF to each BAC

**complete_hit**—Yes if the ORF length and the sum of all its hits to the BAC closely match (indicates full genes on a BAC)

**Multiple gene hits**—Indicates the ORF hits more than one BAC or multiple locations within a BAC

**minBAC/maxBAC**—Rough location of the ORF on the BAC

**BAC**—BAC contig/scaffold name the ORF hit

**Other BAC**—Name of other BAC contigs/scaffolds the ORF hit

**rough identity**—Average identity of each region of the ORF with the BAC region it BLASTed to (not corrected for region size)

**BAC_block**—Manual subdivision of BAC into regions with independent ORFs mapping to them.

**Tabs**

**Summary**—Summarises all combinations of ORF-BAC hits

**Complete, full mapping**—Information and location of complete ORFs, fully mapping to BACs, indicating likely functional transcripts on the BAC contigs/scaffolds

**Complete, truncated mapping**—Information and location of complete ORFs mapping to BACs for only a subset of their length, indicating likely truncated genes on the BAC assembly.

**Remaining tabs**—provide detailed BLAST information per BAC contig/scaffold, that is summarized in the preceding tabs.

**Supplementary File S5.** The aminoacid sequence of predicted genes on the BACs. The names of predicted genes with the same sequence have been combined.
